# What’s wrong with evolutionary biology?

**DOI:** 10.1007/s10539-016-9557-8

**Published:** 2016-12-20

**Authors:** John J. Welch

**Affiliations:** Department of Genetics, University of Cambridge, Cambridge, CB23EH UK

**Keywords:** Adaptation, Extended evolutionary synthesis, Neo-Darwinism, Inclusive fitness

## Abstract

There have been periodic claims that evolutionary biology needs urgent reform, and this article tries to account for the volume and persistence of this discontent. It is argued that a few inescapable properties of the field make it prone to criticisms of predictable kinds, whether or not the criticisms have any merit. For example, the variety of living things and the complexity of evolution make it easy to generate data that seem revolutionary (e.g. exceptions to well-established generalizations, or neglected factors in evolution), and lead to disappointment with existing explanatory frameworks (with their high levels of abstraction, and limited predictive power). It is then argued that special discontent stems from misunderstandings and dislike of one well-known but atypical research programme: the study of adaptive function, in the tradition of behavioural ecology. To achieve its goals, this research needs distinct tools, often including imaginary agency, and a partial description of the evolutionary process. This invites mistaken charges of narrowness and oversimplification (which come, not least, from researchers in other subfields), and these chime with anxieties about human agency and overall purpose. The article ends by discussing several ways in which calls to reform evolutionary biology actively hinder progress in the field.

I’ve been wondering why so many evolutionary biologists are unimpressed by my idea… and I think the problem goes back to Newton (advocate of the Extended Evolutionary Synthesis)

## Discontent and laundry lists

A dispiriting thing about working in evolutionary biology is the steady stream of claims that the field needs urgent reform. These critiques are too numerous to cite, but representatives include Waddington (1957), Moorhead and Kaplan (1966), Ho and Saunders (1984), Gould (1980, 2002), Pigliucci and Müller (2010), and Laland et al. (2014).

These critiques differ greatly from one another; indeed, their conclusions range from the undeniable (“new concepts and empirical findings […] may eventually force a shift of emphasis”; Pigliucci 2007), to the more robust (“It’s wrong like phrenology is wrong. Every major tenet of it is wrong”; Lynn Margulis quoted in Kelly 1994, p. 470). Nevertheless, there are some good reasons for considering the discontent as a whole.

First, some of the critics themselves recognise a shared enterprise, with conferences or multi-authored volumes united solely by the participants’ discontent with current practice. The result is often “laundry lists” of ideas or observations which the field is urged to incorporate or emphasise, but which have little or nothing in common with each other.^1^ The only certainty is that *something* needs to change (Pigliucci 2007; Chorost 2013; Pennisi 2016).

Second, irrespective of the content of the individual critiques, the sheer volume and persistence of the discontent must be telling us something important about evolutionary biology. Broadly speaking, there are two possibilities, both dispiriting. Either (1) the field *is* seriously deficient, but it shows a peculiar conservatism and failure to embrace ideas that are new, true and very important; or (2) something about evolutionary biology makes it prone to the championing of ideas that are new but false or unimportant, or true and important, but already well studied under a different branding.

This article will argue for possibility (2). It will suggest that a few distinct and inescapable properties of evolutionary biology make the field highly likely to attract discontent, regardless of whether the criticisms have any merit.

The aims of the article are therefore limited in two major ways. First, to explain the volume of the discontent, critiques will be painted with a broad brush, to bring out some common features. This is not a substitute for engaging properly with any individual argument. Second, the article will ignore factors common to most of us in most academic subjects, such as self-promotion and the quest for “impact”.^2^ As Kitcher (2000, p. 30) has argued, all scientific controversies entail the construction of “career niches”, but the focus here is on features that are peculiar to evolutionary biology.

## The problems

Some problems for evolutionary biology are caused by the basic characteristics of life. Living things evolved from one or a few common ancestors, but are now characterized by their enormous abundance, variety and complexity. Each is the result of historical processes involving contingencies of distinct kinds (Lenormand et al. 2009), sometimes including one-off events, which might have been highly improbable, but which had profound consequences.

Some banal practical problems are caused by the sheer scope of evolutionary biology. Nobody can hope to read enough of the relevant literature, which means that ideas rightly rejected in one sub-discipline can be rediscovered, or warmed over in others (if the *Drosophila* people aren’t impressed, then you can always try the clinical virologists, or the vertebrate palaeontologists, or the biological anthropologists, etc.), and also makes it almost inevitable that key terms will be used in importantly different ways (as with “adaptation”, “conflict”, “environment”, “epigenetics”, “evolution”, “fitness”, “gene”, “group selection”, “heritability”, “phenotype”, “relatedness”, “selfish”, “species”, etc.; Dawkins 1982, 2004; Maynard Smith 2001; Griffiths and Stotz 2006; West et al. 2007; Haig 2012; Rousset 2015). By confusing these senses, it is easy to make uncontroversial claims sound exciting; this may happen most often with the term “random mutation” (Waddington 1957; Bateson 1958; Laland et al. 2011; Martincorena and Luscombe 2012).^3^

Second, new data appear at a very rapid rate, particularly, in recent years, from molecular biology. This creates the misleading impression that new conceptual frameworks must also be required,^4^ and that valid research programmes are somehow out of date (leading, e.g. to behavioural ecologists feeling compelled to do metabolomics). Third, the scope means that authors are drawn to criticize evolutionary biology when their interests and expertise lie elsewhere. This is often in some branch of human psychology, and philosophers of very different stripes have been accused of mischaracterizing the whole of evolutionary biology solely to bolster a theory of semantic content (Wouters 2005; Rosenberg 2013).

The characteristics of life also make it easy—all too easy—to collect data whose implications seem revolutionary. This is true in two distinct ways. First, all generalizations in biology, from the colour of swans to the misnamed central dogma (Crick 1988), have exceptions (Beatty 1995; Rosenberg 2013), and so it will often be straightforward to make observations that contradict any well-established generalization. Such observations may be truly novel, without being “important” in any other sense. Second, it is straightforward to identify factors that have been little discussed in the biology literature, but which have had a major influence on the evolutionary outcomes in some lineage or lineages. Off the top of my head, “red things” are common in many ecosystems, and are little discussed as a class in the biological literature. However, removing red things, doubling their number, or changing their colour would change the outcomes of evolution in many cases. In this sense, it is easy to show that “Red Things are an important and neglected factor in evolution”. Similar arguments could be made for “gravity”, “burrowing”, “oxidative damage”, “noses”, “histone modification”, etc.

The characteristics of life also guarantee that the explanatory frameworks of evolutionary biology will be disappointing to some. Disappointment is caused partly by the sheer complexity of life’s dynamical processes. This means that our predictive power will always be weak in certain ways, and that our descriptions of evolution will always be enormous simplifications. This isn’t a counsel of despair: while all models are wrong, some are useful (Box 1979; Strevens 2008). Nevertheless, answers to several types of straightforward question must always be incomplete^5^ (Mozley 1884, pp. 396–7; Tinbergen 1963; Strevens 2008), and it will always be easy for critics to claim—correctly—that “things are more complicated”, particularly if they don’t specify their own explanatory goals.

Disappointment also stems from the variety of life. When claims or methods aim at a high generality, they must appeal to common features of living things, and these are few and often abstract. For example, population genetics achieves high generality, since importantly similar processes of genome replication characterize all animals, plants, fungi, protists, bacteria, archaea and viruses. But people interested in these organisms may not be interested in changes in allele frequencies (see Lynch 2007, Ch. 13).

A more widely appreciated feature of evolution is the appearance and spread of conspicuous novelties, “like nothing the world has ever seen before” (Wagner 2015; see also Mayr 1960; Laubichler 2009; Wagner 2014). There are time-honoured ways of studying individual novelties, using various forms of “lineage explanation” (Mayr 1960; Calcott 2009). But a collection of detailed reconstructions is not a theory; and it is not a surprise, or a criticism, that the most interesting and ambitious theories of evolutionary novelty, are both restricted in scope (applying, for example, to a small subset of traits in a minority of organisms: the multicellular eukaryotes), and make their general claims at quite a high level of abstraction (e.g. Wagner 2014, 2015).

Of course evolutionary biology does have a very general and powerful idea. But the theory of natural selection causes additional problems. One problem is its deceptive simplicity (Huxley 1887, p. 197); it is an idea that we all think we understand, but which continues to divide experts (e.g. Lewens 2010; Pence and Ramsey 2013). The simplicity is deceptive in a second sense: our intuitions about natural selection are often very poor. For example, nature is full of “traits whose complexity makes it difficult to see how they can be accounted for by normal natural selection” (Papineau 2005), and so it is tempting to assume that some factor, neglected by current evolutionary thinking, must also have played a “creative role”. This argument from our ignorance stems from Mivart (1871, e.g. Chs. 2 and 4), but it is not restricted to creationists (e.g. Waddington 1960, Ch. 9; Papineau 2005; Nagel 2012; see also Orr 2013).

Finally, something about our attitude to the past, and to the natural world (Plumb 1969; Thomas 1983), makes us demand from evolutionary biology some special kinds of impact (Maynard Smith 1988). These demands are sometimes fairly concrete, e.g. for biological facts to underpin moral theories (Waddington 1960; Rosenberg 1990; MacIntyre 1999; Wilson 2009), but are often much vaguer demands to help us feel “at home in the universe”, or provide us with moral uplift (Waddington 1960, Ch. 9; Saunders 1994, 2003; Kauffman 1995; Jacquet 2005). For authors who make such demands, natural selection causes problems, not only because it is mindless and amoral, but because it can seem downright immoral. For example, Saunders (2003) writes “there is a further danger, as well. Darwinist explanations inherently invoke selfishness and greed as the most important driving forces”. This isn’t true, and even Darwin’s own emphasis on “struggle” probably rests on a mistake (Lewens 2010), but there *is* a very weak sense in which natural selection involves competition, and there is a lot of research on “conflicts”.^6^

## The study of adaptive function

The problems discussed above have no common thread, and they apply widely in evolutionary biology. However, they coalesce in a special way for one research programme: the study of adaptive function. The goal of such research is not a precise description of evolutionary change. Instead, it aims for a strong account of phenotypic function, which is linked to a partial account of why those phenotypes exist.^7^ Research of this kind is found across biology, because traits of all kinds might be adaptations (Maynard Smith 1978; Mayr 1983), but it has attracted most attention in behavioural ecology (Tinbergen 1963; Grafen 1991, 2007; Cuthill 2009).

Such research uses ideas related to optimization, including tools from engineering and economics, and often represents evolution in terms of (imaginary) agents with (imaginary) agendas. To see why this might be useful, consider leaf insects of the genus *Phyllium*, and butterflies of the genus *Anteos*. These genera both contain leaf mimics, which look quite similar, and probably do so for similar reasons. These reasons are adaptive rationales, and they do not imply any similarity in the ontogenies of the genera, and nor in the details of their evolutionary histories (Dennett 1995, p. 21; Papineau 2010). Nevertheless, hypotheses about these reasons can be tested (e.g. from morphology alone, we might predict that certain resting sites will be favoured in a controlled choice experiment; and we might predict that both genera live in habitats that contain similar looking leaves, and visually-guided predators that might dine happily on insects, but not on leaves). Identifying “agendas” is necessary if we want to distinguish the proper function of the mimicry (camouflage from predators), from accidental byproducts (attracting wildlife photographers) and from malfunctions (attracting herbivores). Identifying “agents” is useful for predicting which parts of the world will act as if in accordance with the agenda. For example, leaves play a crucial role in explaining the presence of leaf mimics, but they are not usefully considered as agents in this case (e.g. we would not expect leaves to evolve leg-like structures to more closely resemble their mimics, nor to direct their growth towards them).^8^

Hypothesizing about adaptive rationales is easy to do badly, and difficult to do well. Furthermore, research tends to focus on cases that require some ingenuity (no Crafoord prizes for explaining why leaf insects look like leaves). Nevertheless, a central goal of the study of adaptation has been to *rein in* functional ascription, restricting its use to cases where it does real explanatory or predictive work (Williams 1966). So while the historical processes that have resulted in adaptations are remarkably diverse, research on adaptive function focuses on a single component of the total change—the optimizing tendency that results from, and *is sometimes defined as* the action of natural selection (Maynard Smith 1978; Grafen 1991, 1999, 2007; Gardner et al. 2011). This is the part of the dynamics that makes it useful to talk about functions. Similarly, the adaptations themselves are remarkably diverse, but the list of agents and agendas is very limited. In most cases, the agents are organisms, and their agenda is to increase their inclusive fitness (Hamilton 1964a, b)—although as Dawkins (1976, 1982) argued, more-or-less the same idea can be recast with genes as the agents, and gene survival as the agenda.^9^ This list of agents should not include everything that influences the evolutionary process (no nests, social groups, epigenetic marks or ecosystems), and the agendas should not include everything that organisms do (Tinbergen 1963; Williams 1966; cf. Eldredge 1995, p. 39–40, and Cummins and Roth 2009).

Of course, the theoretical machinery is only needed to understand difficult cases, and in practice, there is a lot of leeway. For example, measures of performance (such as foraging success or steadiness of gait) are often used as proxies for inclusive fitness (Arnold 1983), and the mode of inheritance is often ignored, by focusing solely on the phenotypes (Grafen 1991). Nevertheless, this research programme rejects claims that water can cause adaptations (Gould and Lewontin 1979), or that ecosystems have functions, in the same sense as do, e.g. eyes (Williams 1966; Dawkins 1982, Ch. 13; Jax 2005; Okasha and Paternotte 2012).

Adaptations distinguish living things from other complex dynamical systems, such as piles of sand, or the weather. Nevertheless, it is clear that methods designed for studying adaptive function won’t be of much use for many evolutionary biologists—including those with other sorts of question about adaptations (e.g. Tinbergen 1963; Coyne et al. 1997). Other biologists might want to understand macroecological patterns (such as the latitudinal species gradient), obtain a more detailed description of the evolutionary history of one particular lineage or trait (e.g. the evolution of feathers, or vestigial eyes), investigate the evolution of apparently maladaptive phenotypes (such as reproductive isolation, or senescence), or selective processes that are unlikely to lead to adaptations (e.g. non-transmissible cancers, or between-clade differences in speciation rates), investigate the evolution of the many “second-order” properties that might influence a population’s future evolution,^10^ or simply quantify the action of natural selection (Maynard Smith and Haigh 1974; Lande and Arnold 1983; Goodnight et al. 1992).

This “non-adaptationist” research includes most work in evolutionary biology, including almost all of population genetics. Here, the aim *is* to describe a dynamical process—allele frequency change—and this implies a stronger focus on genetic drift (Wright 1967; Kimura 1983), and a different picture of natural selection. For population geneticists, natural selection is a quantifiable bias in the transmission of alleles between generations. This involves no imaginary agency, and might not lead to anything being optimized. Indeed, it is easy to write down simple models that appear to act as counterexamples to any proposed maximand (Felsenstein 2000).

This seems paradoxical, because population genetics is often said to *underpin* research on biological adaptation—and it does (e.g. Wright 1967, pp 254–255). But the underpinning is subtle (e.g. Maynard Smith 1978; Charlesworth 1990; Eshel 1996; Hammerstein 1996; Grafen 2007; Rousset 2015; Lehmann et al. 2015), full of caveats (as it must be in a world full of maladaptation), and also, in certain ways, contingent: other dynamical processes could do a similar job (Dawkins 2004; Gardner 2011; Godfrey-Smith 2011). The relationship is close for empirical reasons: there just aren’t many (or any?) examples of engineering or agency-type thinking doing useful work in biology, without natural selection acting on genomic variation having played a role. So the two research programmes are closely connected, and they often interact in fruitful ways (e.g. Hinde et al. 2010; Barrett and Hoekstra 2011), but their goals, and pictures of natural selection, remain distinct.

## Critics of research on adaptive function

Several different critiques of evolutionary biology make more sense if understood, at least in part, as failures to appreciate the distinctive goals of research on adaptive function.^11^ For example, there has been a recent call to abandon the most successful addition to evolutionary biology in the last few decades: W. D. Hamilton’s inclusive fitness theory (IFT), sometimes called “kin selection”^12^ (Hamilton 1964a, b). These critics (Nowak et al. 2010; Allen et al. 2013) have argued that:

(i) IFT often gives a description of allele frequency change that is inadequate and/or inaccurate, particularly when there are “complex interactions”.
(ii) IFT has made few predictions that are truly quantitative.
(iii) IFT is never necessary, because it is always possible to give a complete description of allele frequency change that makes no mention of IFT.

The critics conclude that IFT should be abandoned, in favour of “standard natural selection theory”. Defenders of IFT (e.g. Gardner et al. 2011; Gardner and West 2014; Rousset 2015) have replied that:

(iv) IFT is a predictive theory of adaptation, which is successful *because* it focusses on a single component of the evolutionary change: the optimizing tendency that characterizes a very broad class of evolutionary histories.
(v) IFT has explained a very large number of biological adaptations that had proven puzzling (e.g. adaptations involving suicidal self-sacrifice).
(vi) Beyond this basic insight, IFT has been a remarkably productive research programme, with a large number of testable, and tested predictions.

Notice that one could agree with all six claims, and diagnose only a difference in research goals.

Arguments very similar to (i)–(iii) have also appeared in other contexts.^13^ While the details differ, the common claim is that research in evolutionary biology oversimplifies the process of evolution, by placing too much emphasis on a limited range of factors (natural selection, unlinked genes, additive effects etc.).

These arguments are familiar, in part, because they are made by two quite different groups of critics. First, there are researchers who are interested in describing evolution, but not very interested in adaptive function. To these researchers, a lot of valid research in evolutionary biology can seem sloppy, inappropriately anthropomorphic, or willfully ignorant of evolution’s complexities. In this case, arguments similar to (iv)–(vi) can be an appropriate response: for researching biological function/malfunction and adaptive rationales, it can be useful or necessary to focus exclusively on natural selection conceived as an optimizing tendency, and on a limited range of agents and agendas (Mayr 1983; Gardner 2013).

By contrast, the second group of critics *dislike* standard theories of adaptive function, and wish to see them undermined. The reasons for this dislike are not always scientific, but involve anxieties about agency and overall purpose, which the theories seem to raise (e.g. Waddington 1960, Chs. 6 and 9; Francis 2004; Church 2007).^14^ The anxieties are easy enough to understand. While naturalistic, the theories superficially resemble a transcendental account of value (they provide criteria for judging behaviours as better or worse, without reference to anybody’s attitudes),^15^ but the values that they superficially endorse are unattractive (the imaginary motives of the imaginary agents are generally base), and in some accounts, the imaginary agents are not even humans. Anxieties can be real, even if they are baseless, and the aims of these critics are best viewed as therapeutic.

To this end, theories of adaptive function can be undermined in any number of ways. A common strategy is to diffuse the imaginary agency more liberally around the system: first, by emphasizing any of the factors that the theories neglect (ignoring the principled reasons for their neglect), and then, by (re)describing the factors using the language of agency (e.g. “natural genetic engineering”, “directed mutation”, “self-organisation”, “Gaia”, “niche construction”), or terms with strong connotations of agency (such as “non-random” or “plasticity”; Woodfield 1976, Ch. 3). The result need not be a genuine alternative theory of function (i.e. an account that might be used to predict or explain anything).^16^ As long as the description of evolution “seems easier to bring into relation with our moral feelings” (Waddington 1960, p. 100), it might help us to behave or feel better. If the imaginary agency is sufficiently diffuse, dynamical systems theory can become a sort of mysticism (e.g. Bateson 1958; Waddington 1960, pp. 98–100; Saunders 1994; Goodwin 2001, Ch. 7; Capra and Luisi 2014).

If both groups of critics misunderstand the study of adaptive function, they do so in radically different ways, and with radically different motivations. Nevertheless, neither group writes in isolation, and each can influence, and be influenced by the other (Laland et al. 2011): the first group becoming convinced that their descriptions of dynamical processes are challenging to orthodoxy, and relevant to Big Questions, and the second group becoming convinced that their nagging doubts about “Neo-Darwinism” have a firm empirical or mathematical basis. In this way, the conviction that laundry list topics are “important” can simply emerge, without anybody being able to explain why.^17^

## Discussion

Claims that evolutionary biology is flawed or importantly incomplete are as old as the field. The criticisms are characterized by their diversity at any given time (as seen in the laundry lists of ideas and observations that are championed by critics; Ho and Saunders 1984; Pigliucci and Müller 2010; Chorost 2013; Rose 2016a), and by their persistence over time (West-Eberhard 2009); there are very strong similarities between the arguments of, say, mid-twentieth century critics (e.g. Waddington 1957, 1960; Bateson 1958), and critics writing over 50 years earlier (e.g. Mivart 1871; Mozley 1884, pp. 396–7), or 50 years later (e.g. Laland et al. 2011, 2014; Teresi 2011; Rose 2016a, b).

This does not mean that the critics are wrong. Both the diversity and persistence of criticism might be explained by intransigence and lack of ambition in the field, by “the intolerance and narrow-mindedness of some of those who advocate [Darwinism]” (Mivart 1871), particularly in its “orthodox”, “ultra” or “hardline” forms (Teresi 2011; Rose 2016a).^18^ However, this explanation is not very plausible. Critics and “novel” findings of all kinds have never lacked attention in evolutionary biology, and this sprawling field could have no means of enforcing conformity to any of its tenets,^19^ even if it could agree on what they were.

It has been argued here that the discontent is better understood as stemming from a few inescapable properties of living things, which lead to disappointment with evolutionary biology, and a nagging feeling that reform must be overdue. It has been further argued that particular discontent stems from misunderstandings and dislike of one well-known subfield: the study of adaptive function, in the tradition of behavioural ecology (Tinbergen 1963; Cuthill 2009). One of the few things shared by the laundry list items is their minor role in theories of adaptive function. Therefore, all can be championed as alternatives to such research (things we that we might, or should, study instead), or as observations that promise to *invalidate* these theories (because they affect outcomes in the world, but are not centre stage in the theories). This subfield is a particular target because it is atypical (most researchers do not share its goals), because it requires a very partial description of evolution (as something like inclusive fitness maximization), and because it uses ideas of apparent purpose and imaginary agency, but in a limited way (to make testable predictions, and not to inspire or dignify).

For all of these reasons, the special criticism directed at the subfield is not surprising, but it is a pity. With its inherent focus on plastic phenotypes (Grafen 1999), and whole organisms (Grafen 2007), its common focus on sociality and cooperation (West et al. 2007), deep roots in ecology (Cuthill 2009), strong ties to developmental biology (Hogan and Bolhuis 2009), agnosticism about the details of inheritance (Grafen 1991; Gardner 2011), and above all, its remarkably productive synthesis of modelling, field observation and experiment, behavioural ecology seems like the sort of science that many critics of evolutionary biology might otherwise embrace.

If the account above can explain some puzzling things, it also has obvious weaknesses. First—and as ever in biology—an attempt to explain a pattern is not to deny the exceptions. This article makes no serious attempt to rebut any single criticism of evolutionary biology, and no attempt at all to restrict what is studied. What *has* been argued is that there can be smoke without fire—that persistent and voluble criticism of evolutionary biology is to be expected, whether or not anything is seriously wrong.

Second, the above account involves some speculation about extra-scientific motives, and this is always foolhardy and offensive. But it may be unavoidable. We do need to explain why ideas are so often hailed as important before they have done much scientific work, and why claims that seem utterly banal (‘things are more complicated’, ‘natural selection doesn’t explain everything’, ‘individuals with the same genotype can have different phenotypes’), might be treated as momentous, vital or urgent. It is also undeniable that a lot of writing about evolutionary biology has its mind on higher things (the same, shopworn collection of topics, from Marx and Spencer, to markets and trolley problems). Evolutionary biology, like history, but unlike other natural sciences, raises issues of purpose and agency, alongside those of complexity and generality (Anonymous 1953; Mount 2016), and so there will always be those who agree with Carr (1964) that methods and explanatory goals cannot and *should not* be separated from political or religious agendas (Maynard Smith 1998, Ch. 5; Rose 2016a). Some may even agree with Bateson (1958), that Waddington’s work on genetic assimilation (showing that environmentally-induced traits can become less sensitive to environmental conditions following allele frequency change) really does have implications for “the battle between non-moral materialism and the more mystical view of the universe”.^20^ It is remarkable, for example, that much of the funding for challenging current practice in evolutionary biology comes from The John Templeton Foundation (Pennisi 2016), which is committed to using science to reveal underlying purpose, and rejecting what Nagel (2012) calls “the Materialist Neo-Darwinian Conception of Nature”. But perhaps this is just history repeating itself as farce: if poetry couldn’t save us, nothing on the laundry lists will either.

If criticism of evolutionary biology is inevitable, why grouse about it? It is easy to habituate to misleading alarm calls (Cheney and Seyfarth 1988), and churlish to complain about peripheral ideas, which, by definition, have little influence on what most scientists do. However, claims that evolutionary biology is misguided or importantly incomplete are not harmless, but actively hinder progress in the field. Indeed, they do so in several ways. First, the claims misrepresent the field to the wider public. It is unfair to use guilt by association—many fine studies are cited on creationist websites—but a field that urgently needs reform *is* a field “in crisis” (Mazur 2010), and when it fails to reform, this lends credibility to claims that scientists are, at best, hidebound and foolish, and at worst, guilty of ideologically-motivated deception (Mazur 2010; Teresi 2011). Such claims find an eager audience among those who reject the scientific consensus on other grounds. For example, Fodor and Piattelli-Palmarini (2010) present a priori objections to (their version of) natural selection, but also include a fairly typical laundry list to add some empirical heft. Chorost (2013) criticized Nagel (2012) for *not* including a laundry list. Second, and within the field, the claims encourage neophilia. This makes us unwilling to build on previous work, to integrate new findings and ideas with existing explanatory frameworks, to replicate published results (Nakagawa and Parker 2015), or to solve the field’s many outstanding problems (Maynard Smith 1977; John 1981). It also distracts attention from the ways in which all biologists can do something genuinely new, such as expanding the range of study organisms. The comparative method (Maynard Smith and Halliday 1979), Krogh’s principle (Krebs 1975), and our ignorance of biodiversity (Nee 2004), all suggest that this is one way that we might usefully extend the field.

## Footnotes

1 E.g., “evolvability, phenotypic plasticity, epigenetic inheritance, complexity theory, and the theory of evolution in highly dimensional adaptive landscapes” (Pigliucci 2007); "modularity, hierarchy, constraints, evolvability, novelty, and emergence, robustness, history, etc." (Laubichler 2009); “evolvability, developmental plasticity, phenotypic and genetic accommodation, punctuated evolution, phenotypic innovation, facilitated variation, epigenetic inheritance, and multi-level selection” (Pigliucci and Müller 2010). Rose (2016a) mentions genetic accommodation, epigenetics, co-operative interactions, group and ecosystem selection, self-organisation, niche construction, behavioural and symbolic transmission, and Marxism. See also Chorost (2013) and Maynard Smith (1988), Ch. 18.

2 “What I tried to say about Steve Gould is that scientists are always looking to find some theory or idea that they can push as something that nobody else ever thought of because that’s the way they get their prestige… they have an idea which will overturn our whole view of evolution because otherwise they’re just workers in the factory, so to speak. And the factory was designed by Charles Darwin.” (Richard Lewontin, cited in Mazur 2010, p. 30). See also Eldredge (1995, p. 56).

3 The claim that mutation is undirected (or “random”) is perhaps the closest thing to a tenet of evolutionary biology. It states that a truly novel environment will not preferentially induce mutations that are appropriate to that environment. This must be distinguished from the claim that the rate and spectrum of mutations might adaptively evolve (e.g. Kimura 1967), and from the falsism that all mutations are equally likely to occur. If directed mutation did happen, it would be very important, and very difficult to explain with current scientific theories; but there are many more published articles about directed mutation than well-documented instances. Miller (1999) makes an explicit connection to miracles.

4 For example, Pigliucci (2007) argued that “one would not expect the original synthesis to be able to address directly the wealth of information emerging from genomics […] and the other new ‘-omics’ sciences”. But it is doing so (e.g., Lynch 2007). The fact that old frameworks are so often applicable to novel data follows directly from their high levels of abstraction.

5 This is clearest when we ask why something didn’t happen in a particular lineage: Why are there so few truly marine insects? Why aren’t there penguins in the arctic? Why don’t pigs have wings?

6 Evolutionary conflicts can arise when interacting biological agents are of different types (i.e. symbionts and hosts, males and females, parents and offspring, nuclear- and mitochondrially-encoded genes, etc.), although in all cases, the outcome might also be mutualism. It can seem as if an adolescent wish to shock—or worse—motivates the focus on nature’s horrors (Kaplan 2009), but this is a canard. Conflicts are of special interest for three prosaic reasons: they are more likely than mutualisms to lead to ongoing evolution; they play an important role in disease (Stearns and Koella 2009); and the widespread *assumption* of shared interests has led to several scientific errors (Williams 1966).

7 I am bracketing several debates here, whose importance for scientific practice can be exaggerated. In short, functional ascription can be indeterminate and change over time, while the presence of a trait can be explained in several different ways, all of which will be incomplete (see, e.g., Tinbergen 1963; Mayr 1997; Cuthill 2009; Perlman 2009). The research described is connected, but not equivalent, to any of Lewens’ (2009) “seven types of adaptationism”.

8 For some purposes, it may be possible to avoid intentional language altogether (Godfrey-Smith 2011), but it is both useful, and *necessary* if we want to explain why organisms look as if they were designed (since apparent design implies imaginary agency). This argues, against Cummins and Roth (2009), that “accounting for some (suspect) intuitions” is a legitimate goal of the theory.

9 The “agent” has several distinct roles in Dawkins’ framework, some of which have no analogue in the organism-as-agent approach, and none of which imply selfishness in any strict sense (Dawkins 1982, 2004; Lloyd 2001; Gardner and Welch 2011; Haig 2012; Bourke 2014). Dawkins’ recasting, of course, motivates the common claim that theories of adaptive function are “reductionist”. This is difficult to take seriously; the research involves imaginary agents.

10 These topics all relate to what Richard Dawkins dubbed “evolvability”, and include the evolution of mutation rates (Lynch 2010), recombination rates (Barton 1995), environmental variance (Hill and Mulder 2010), pleiotropy (Wagner and Altenberg 1996), mating systems etc. Such properties of the genetic system can all evolve under the action of natural selection, but are not easily studied with optimization-based approaches (e.g. Grafen 2007). Indeed, this is the area of evolutionary biology where unsupported claims about adaptive function are most common (Lynch 2007, Ch. 13), with failures to distinguish between simple descriptions (‘introns are sometimes involved in the adaptive evolution of gene regulation’) and much stronger statements (‘introns evolved to facilitate adaptive changes in gene regulation’).

11 The failure of understanding is clearest in claims that the research programme is empty: that by manipulating the Price equation (a mathematical identity), and relegating factors to the transmission term, researchers make claims that are unfalsifiable (Pigliucci 2007; Cummins and Roth 2009; Allen et al. 2013). This echoes the old criticism that natural selection is a tautology (Maynard Smith 1969), and takes no account of how predictive theories of adaptation are actually used.

12 My account of this debate is simplified, and I ignore criticisms that do not put the opposing case at its strongest. For example, Allen et al. (2013) make the potentially important criticism that IFT confuses correlation and causation, but the validity of their claim will not be possible to judge until they incorporate the necessary class structure into their IFT analyses.

13 For example, (i) and (ii) echo Mayr’s (1959) attack on “bean-bag genetics” (which itself echoes Waddington 1957, pp. 59–60), and which Mayr (1983) connects to *part* of Gould and Lewontin’s (1979) attack on “adaptationism”. Saunders (1994), echoes (iii) when he argues that “there seems no particular reason to invoke natural selection" to describe his model, while Wood and Coe (2007) show that there are good reasons.

14 Waddington (1960) disliked theories where “mutation appears as an external force, to which the organism passively submits” (p. 88), or where evolution “just happened to go the way it did, but for no ascertainable reason” (p. 89). Rose (2016a, b) summarizes his own “main argument” thus: “redefining evolution as ‘a change of gene frequency in a population’ is a reductionism too far, depriving living organisms of playing any part in their own destiny”.

15 This relates to a more serious question: whether standard accounts of adaptive function undermine *all* realist theories of value (Street 2006). Realist theories have been defended by appealing to niche construction (Rouse 2015), or to group selection (Okrent 2016), or to something as yet unknown to science (Nagel 2012). Waddington, also concerned with this issue (1960, pp. 98–100), made a case for all three processes, and for genetic assimilation, although his arguments are embryonic (see Chs. 6 and 9).

16 The major exception is “old group selection”, with its claim that adaptations often function for the good of the group (Williams 1966; West et al. 2007; Okasha and Paternotte 2012; Bourke 2014; see also Waddington 1960, Ch. 6).

17 There is ambiguity even in the most serious and substantial arguments. For example, Laland and Sterelny (2006) argue that existing research on extended phenotypes, and other types of organism-induced environmental change, should be renamed as "niche construction", so that the work can be recognised as “part of a new and valuable general framework”. This is, at least in effect, a request to shift emphasis away from the study of adaptive function, and towards more detailed dynamical descriptions of particular lineages (see also Laland et al. 2011; Gardner 2013), but there are also phrases that read like emotive requests to diffuse the agency. For example, the authors express regret that “the active agency of beavers […] acting as codirectors of their own evolution […] currently goes unrecognized”, but do not show how attributing imaginary agency in a new way might lead to novel predictions. They state that the standard approach "misses part of the causal story" (which is true of all possible approaches), but identify no scientific errors that have resulted from the approach, and rely on unsupported claims about what “most biologists simply assume”. None of this would have mattered to Waddington (1960), who had non-scientific reasons for elevating the beaver to codirector, and focusing on cybernetic feedbacks (Chs. 6 and 9).

18 Others have argued that biologists must suffer from “cyclic amnesia” (West-Eberhard 2009), or a failure to understand causation (Waddington 1960, Ch. 9; Laland et al. 2011).

19 For a remarkable, but atypical attempt, see (The Editors 2016).

20 The fact that the laundry list items play little role in theories of adaptive function is another way of saying that they cannot explain apparent design. This may explain why so many appear in natural theology (e.g. Bateson 1958; Bowler 1989, e.g., p. 259; Crawford 2005).
